# From skin to heart: a state-of-the-art perspective on psoriasis-driven cardiovascular comorbidities

**DOI:** 10.3389/fimmu.2026.1923150

**Published:** 2026-09-09

**Authors:** Alex Spitilli, Davide Ferrari, Eva Reali

**Affiliations:** 1 Department of Life Sciences and Biotechnology, University of Ferrara, Ferrara, Italy; 2 Department of Translational Medicine, University of Ferrara, Ferrara, Italy

**Keywords:** atherosclerosis, chronic inflammation, psoriasis, skin-vascular axis, vascular remodeling

## Abstract

Psoriasis is a chronic inflammatory disease associated with major adverse cardiovascular events that persist after adjustment for traditional risk factors. The cardiovascular phenotype extends beyond coronary atherosclerosis to involve microvasculature, carotid arteries, and a prothrombotic state. This review integrates preclinical and clinical evidence to delineate the mechanistic relationship between the inflamed skin and the vessel wall. Three converging mechanisms emerge by which skin-derived inflammation reaches the vasculature. The first is a cytokine and matrix-remodeling mechanism. This potentially involves both IL-17A/IL-17F, acting via lysyl oxidase-dependent collagen crosslinking to entrap lipoproteins, and cutaneous IL-6, which serves as a link between skin inflammation and the prothrombotic state of the arterial wall. The second is a T cell-driven mechanism, in which skin-primed effector cells with vascular tropism act on the arterial endothelium and accumulate in atherosclerosis-prone vessels. The third is a myeloid and oxidative mechanism, fueled by inflammatory hematopoiesis and macrophage reprogramming. Across murine models, the cardiovascular phenotype appears more closely linked to the chronicity of inflammation than to the intensity of individual flares. Acute models generally fail to accelerate atherosclerosis, whereas genetic models that sustain chronic inflammation such as the K14-Rac1V12, KC-Tie2 and Card14-mutant models are associated with accelerated atherogenesis. Consistently, in patients, disease duration emerges as an independent predictor of cardiovascular events.

## Introduction

Psoriasis is a chronic, inflammatory disease involving skin, nails, and/or joints in which the IL-23/IL-17 axis sustains a self-perpetuating cycle of keratinocyte hyperproliferation and dermal infiltration of T cells and myeloid cells (1–3). The same immune pathways also operate in atherosclerosis, and through this overlap the chronically inflamed skin behaves as a persistent source of proatherogenic signals (4).

Large population studies have documented a higher incidence of myocardial infarction, cardiovascular mortality and major adverse cardiovascular events in patients with psoriasis compared with the general population, with the strongest risk observed in severe disease (5–7). A recent meta-analysis of 21 observational studies, comprising more than 770,000 patients with psoriasis, quantified this association, reporting an increased risk of atherosclerotic cardiovascular disease and myocardial infarction (hazard ratios of approximately 1.2) (8). The cardiovascular phenotype is not confined to coronary atherosclerosis: it includes coronary microvascular dysfunction, an increased burden of subclinical carotid disease, myocardial deformation, a prothrombotic propensity documented at the molecular and cellular level, and a higher prevalence of cardiac arrhythmias, particularly atrial fibrillation (9, 10). The association with cardiovascular events persists after adjustment for traditional risk factors, and conventional risk algorithms fail to capture the imaging-defined coronary plaque burden carried by these patients (11). The duration of psoriasis has emerged as an independent predictor of cardiovascular events, an effect that persists after adjustment for traditional risk factors and is paralleled by a duration-dependent increase in vascular inflammation, indicating that the relevant pathological process may reflect cumulative inflammatory exposure rather than transient activation (12).

The concept of a “psoriatic march”, in which chronic cutaneous inflammation has been proposed to propagate to the cardiovascular system through systemic immune and metabolic mediators, was introduced more than a decade ago and has since attracted progressive mechanistic investigations (13).

Direct support for causal contribution of inflammation to human cardiovascular disease was provided by the CANTOS trial, in which IL-1β neutralization with canakinumab reduced major adverse cardiovascular events independently of LDL-cholesterol lowering (14).

The immunological circuits that translate skin inflammation into cardiovascular disease, however, remain only partially defined. The IL-23/IL-17 axis is a central driver of the cutaneous phenotype, yet the extent to which IL-17 blockade translates into cardiovascular benefit in patients is less well established than its dermatological efficacy, raising the possibility that additional pathways operating outside the canonical IL-17 circuit contribute to the cardiovascular phenotype.

In this review we integrate the preclinical and clinical evidence accumulated over the past ten years to delineate the mechanistic architecture of the cardiovascular comorbidities associated with psoriasis.

## Search strategy

The evidence analyzed in this review was identified through a structured PubMed search designed to retrieve original preclinical and clinical research on the mechanistic relationship between psoriasis and cardiovascular disease. The search was conducted on April 8, 2026, and the identification, screening and selection of records followed the Preferred Reporting Items for Systematic Reviews and Meta-Analyses (PRISMA) framework. The disease term was combined with cardiovascular and vascular imaging outcomes and restricted to publications from January 1, 2016 onwards. Records classified as reviews, systematic reviews, meta-analyses, editorials, comments, letters, news items or case reports were excluded at the query level to focus retrieval on original data. The full search string was as follows:

(psoriasis[MeSH Terms] OR psoriasis[tiab]) AND (atherosclerosis[MeSH Terms] OR atherosclerosis[tiab] OR “coronary artery disease”[tiab] OR coronary[tiab] OR “vascular inflammation”[tiab] OR “coronary artery calcium”[tiab] OR CAC[tiab] OR “coronary computed tomography angiography”[tiab] OR CCTA[tiab] OR “carotid intima-media”[tiab] OR cIMT[tiab] OR myocardial infarction[tiab] OR stroke[tiab] OR MACE[tiab]) AND (“2016/01/01”[dp]: “3000”[dp]) NOT (review[pt] OR systematic review[pt] OR meta-analysis[pt]) NOT (review[pt] OR systematic review[pt] OR meta-analysis[pt] OR editorial[pt] OR comment[pt] OR letter[pt] OR news[pt] OR case reports[pt]).

**Figure 1:**
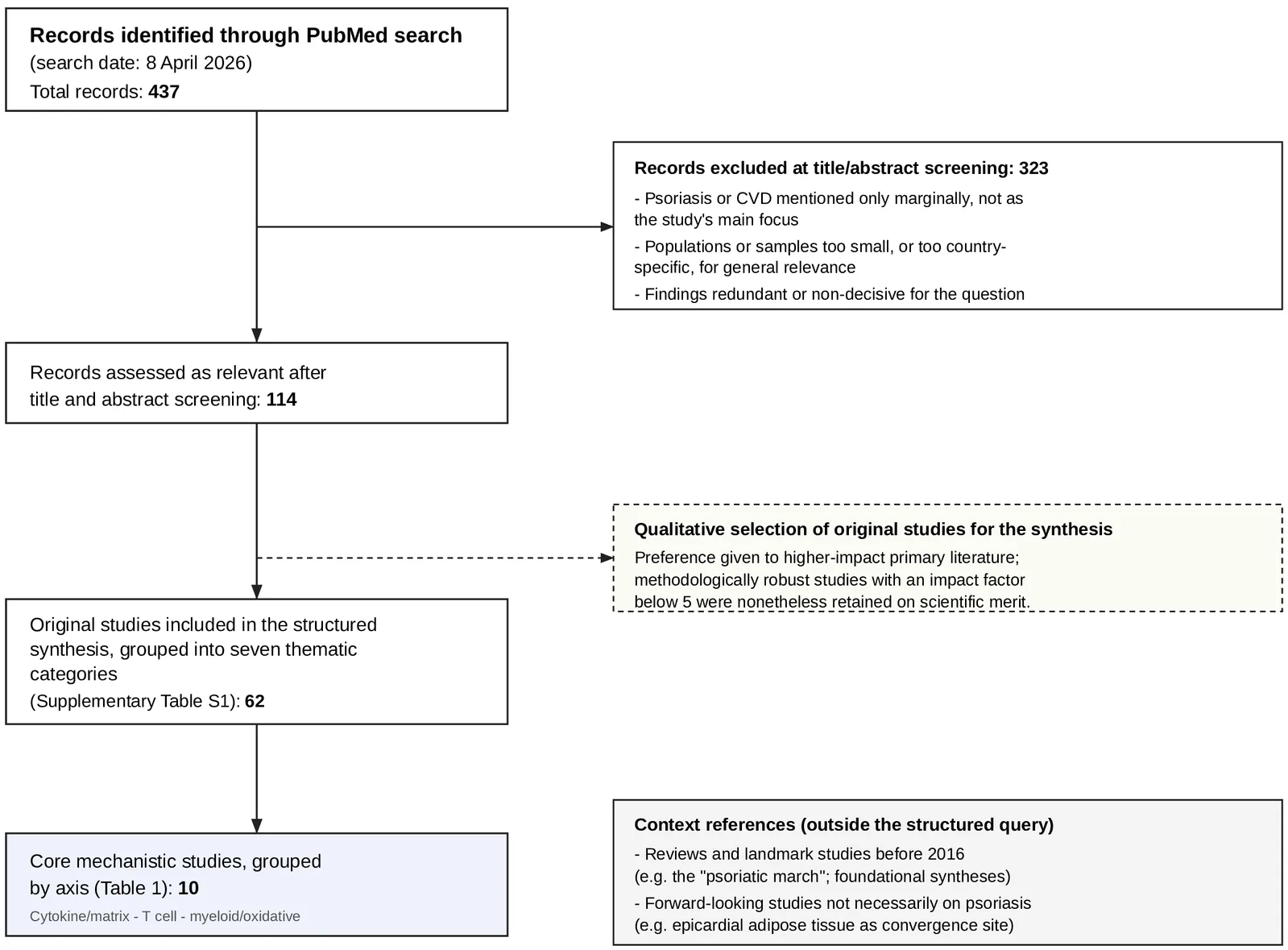
Identification and selection process of the studies included in the review, from the 437 records retrieved through the structured PubMed search of 8 April 2026.

The search returned 437 records. Screening of titles and abstracts removed the records that, although matching the search terms, did not address the mechanistic, vascular or imaging interface between psoriasis and cardiovascular disease, samples too small to support conclusions, or contributed only redundant or non-decisive findings; 114 records were retained as relevant. From these, 62 original studies were selected for the structured synthesis and are summarized, grouped by thematic category, in **Supplementary Table 1**. Priority was given to higher impact primary literature where available. Importantly, several studies published in journals with an impact factor below 5 were nonetheless retained based on their robustness and mechanistic relevance. Among the selected articles, ten proved central to the structure and interpretation of the review and are presented in **Table 1**, grouped by mechanistic axis: cytokine and matrix remodeling, T cell-driven, and myeloid/oxidative mechanisms. The selection flow is shown in **Figure 1**.

**Table 1:** Selected studies illustrating the three mechanistic axes that link psoriasis to CVD.

Ref.	First author, year	Study type	Model/design	Key findings
Axis 1 cytokine signalling and matrix remodelling (IL-17A/F, IL-6, LOX)				
15	Huang LH, 2019	Preclinical	IMQ C57BL/6 mouse	IL-17 induces LOX-mediated interstitial entrapment of HDL and LDL in skin and aorta; reversed by anti-IL-17 or by BAPN.
16	Schüler R, 2019	Preclinical	Models of graded severity: two IL-17A-overexpressing lines (K14-IL-17A; CD11c-IL-17A) and the acute IMQ model	Serum IL-17A concentration encore the severity of vascular dysfunction across the three models; anti-IL-17A improves endothelial function.
19	Nakanishi T, 2024	Preclinical	Kcasp1Tg crossed with IL-17A/F KO	Aortic arteriosclerotic remodelling requires deletion of both IL-17A and IL-17F, indicating a non-redundant contribution of IL-17F beyond IL-17A; the cellular source of IL-17F was not directly determined.
24	Wang Y, 2016	Preclinical	KC-Tie2 mouse with or without IL-6 KO	Genetic deletion of IL-6 restores thrombotic occlusion time towards control levels without altering skin score, identifying skin-derived IL-6 as a critical link to the prothrombotic state.
Axis 2 skin-primed T cells with vascular tropism				
21	Casciano F, 2025	Preclinical + human PB phenotyping	Recurrent IMQ C57BL/6 mouse + 36 psoriasis patients	Repeated IMQ cycles expand CXCR3+ highly differentiated memory T cells systemically; aortic *Icam1*, *Vcam1*, *Ccl2* and *Olr1* upregulated.
26	Baral I, 2025	Preclinical + human cohort	IMQ-ApoE−/−, IL-23-ApoE−/− and Card14ΔE138-ApoE−/− mouse on HFD; human cohort with ASCVD	Th9/IL-9R/STAT3 axis acts directly on arterial endothelium; chronic Card14 model reproduces atherosclerosis whereas acute IMQ does not; anti-IL-9 reduces aortic plaque area; expanded Th9 cells associate with ASCVD in patients.
Axis 3 myeloid reprogramming and oxidative stress				
20	Schaller T, 2023	Preclinical	K14-IL-17Aind/+ × PhAM mouse (photoconversion-based cell tracking)	Proinflammatory neutrophils reaching the aortic wall originate from the bone marrow, not from the psoriatic skin; ROS-mediated vascular inflammation.
28	Baumer Y, 2018	Preclinical	K14-Rac1V12 × ApoE−/− × HFD mouse	SOD2 expression reduced by approximately 60% in psoriasis macrophages; mitochondrial ROS accumulate and drive foam cell formation; on an atherosclerosis-prone background, atherogenesis is accelerated of approximately 7-fold. SOD2 activity reverses the proatherogenic phenotype.
29	Dong C, 2025	Preclinical (scRNA-seq + mouse)	Patient skin scRNA-seq integrated with IMQ mouse	Platelet GPIb upregulated in psoriatic skin in a perivascular distribution, correlated with PASI; engages monocyte CD11b to drive M1 polarisation; bidirectional skin-atherosclerosis circuit.
69	Nakamura Y, 2024	Translational (human + mouse)	Human monocyte-derived macrophages *in vitro* + mouse atherosclerosis	LL-37 enhances LDL uptake by human macrophages via LDLR, SR-B1 and CD36; recombinant LL-37 accelerates plaque formation in murine atherosclerosis.

## IL-17 in psoriasis-associated cardiovascular disease: from matrix remodeling to lipoprotein entrapment

It is well established that the IL-23/IL-17 axis is a key driver of skin inflammation in psoriasis (1–3). However, recent literature indicates that the effect of IL-23/IL-17 activation might extend beyond the skin, up to the vascular tissue and cardiovascular compartment (5–7, 13).

Topical imiquimod (IMQ), applied to wild-type C57BL/6 mice for 14 days, produced psoriasis-like skin lesions. In the draining lymph nodes, these lesions programmed IL-17-producing T cells that subsequently migrated to distant skin and to the arterial wall (15). At these sites, IL-17 drove the accumulation of collagen, which was then stabilized by lysyl oxidase (LOX)-mediated crosslinking into an insoluble matrix that remodeled the interstitial space and entrapped large molecules, including high-density (HDL) and low-density lipoproteins (LDL). Most importantly, this process was reversed by depletion of CD4^+^ T cells, by neutralization of IL-17, or by inhibition of LOX-mediated collagen crosslinking with β-aminopropionitrile (BAPN), which restored HDL transit to baseline levels. Experimental psoriasis also increased vascular stiffness, and, in hyperlipidemic apolipoprotein E-deficient mice, it augmented the atherosclerotic burden, an effect that was attenuated by IL-17 neutralization (15).

The central role of IL-17A in the vascular pathology associated with psoriasis was shown in another study by using three murine models with different severity index (16): (i) K14-IL-17Aind/^+^ mice with keratinocyte-specific IL-17A overexpression and an early-onset severe phenotype; (ii) CD11c-IL-17Aind/ind and CD11c-IL-17Aind/^+^ mice overexpressing IL-17A in CD11c^+^ cells with delayed-onset and moderate disease; and (iii) classical imiquimod (IMQ) acute model. Across all three settings, vascular dysfunction was correlated with the plasma levels of IL-17A as well as with Ly6C^+^Ly6G^+^ myeloid cell infiltration into the aortic wall. Anti-IL-17A treatment, in the moderate-severity model, showed attenuation not only of the cutaneous lesions but also of the peripheral oxidative stress, proinflammatory cytokine levels, and endothelial tissue inflammation (16),. The proatherogenic role of this cytokine is further supported by two independent studies in apolipoprotein E-deficient mice, in which blockade of IL-17A reduced atherosclerotic lesion development and aortic macrophage content (17, 18).

The relative contribution of IL-17A and IL-17F to the extracutaneous manifestations was investigated using the Kcasp1Tg model, in which the overexpression of human caspase-1 in keratinocytes drives chronic severe dermatitis and systemic amyloidosis. To assess the contribution of IL-17, mice were crossed with IL-17A^−/−^, IL-17F^−/−^, or double-knockout IL-17AF^−/−^ mouse strains (19).

The results revealed a differential requirement for the IL-17A and IL-17F cytokines showing that systemic amyloidosis was primarily IL-17A-dependent whereas the arteriosclerotic changes in the abdominal aorta required deletion of both IL-17A and IL-17F for their resolution. This distinction is important because it indicates that IL-17A and IL-17F are not functionally redundant. The finding identifies a skin-to-vessel mechanistic link in which IL-17F contributes to the vascular phenotype beyond IL-17A, although the cellular source of IL-17F in this model was not directly determined (19).

Another study used a K14-IL-17Aind/+ model overexpressing IL-17A in keratinocytes, crossed with PhAM (photoactivatable mouse) reporter mice. These mice express the photoconvertible protein mito-Dendra2, which switches irreversibly from green to red fluorescence upon photoconversion, allowing cells labeled at a defined anatomical site to be tracked as they migrate *in vivo*. Using this system, the proinflammatory neutrophils reaching the aortic wall were shown to derive from the bone marrow rather than from the psoriatic skin (20). These bone marrow-derived myeloid cells exhibit heightened oxidative burst and elevated expression of *Cxcl2* and *S100a9* both in the skin and in the aorta, pointing to inflammatory hematopoiesis, rather than the psoriatic skin, as the origin of the vascular myeloid infiltrate in IL-17A-driven psoriasis.

The hypothesis that recurrent episodes of skin inflammation progressively build a pool of pathogenic T cells with vascular tropism has recently been tested in a model of recurrent psoriasis (21). The results showed that the systemic accumulation of memory T cells with a highly differentiated phenotype was paralleled by an upregulation of endothelial dysfunction genes (*Icam1*, *Vcam1*) and an increase in vascular inflammation markers (*Ccl2*, *Olr1*), in aortic tissue. A trend towards increased expression of *Cxcl10*, the ligand of CXCR3, was also observed, providing a potential chemotactic link between the expanded subset of CXCR3^+^ T cells and vascular inflammation (22, 23).

## Beyond IL-17: parallel cytokine axes and macrophage reprogramming

Evidence of IL-6 as a mediator between skin inflammation and thrombosis was established using the KC-Tie2 model, in which ectopic expression of the angiopoietin receptor Tie2 in keratinocytes produces a chronic, spontaneous psoriasiform phenotype with a prothrombotic state, manifested as shortened times to thrombotic occlusion (24). When IL-6 was genetically deleted in this model (KC-Tie2×IL-6−/−), the time to thrombotic occlusion was restored towards control levels despite persistence of the cutaneous inflammation, identifying skin-derived IL-6 as a critical link between psoriasiform inflammation and the prothrombotic state. This observation acquires translational relevance since IL-6 and its receptor are elevated in psoriatic skin, and the IL-6 receptor is detected in human coronary atheroma, providing a plausible molecular bridge between skin inflammation and vascular disease (24). At the population level, circulating IL-6 has been consistently associated with cardiovascular risk (25).

Importantly, an immune axis distinct from IL-17 and TNF-α was identified through a multi-level translational approach focusing on T helper 9 (Th9) cells (26). Three complementary murine models of psoriatic atherogenesis were employed, all on an ApoE^−/−^ background and fed with a high-fat diet to drive plaque formation. The IMQ-ApoE^−/−^ model relied on cyclic topical imiquimod applications to elicit an acute, innate-driven psoriasiform inflammation. In the IL-23-ApoE−/− model, intradermal IL-23 injection was used to drive psoriasiform skin inflammation. The Card14ΔE138-ApoE−/− model carries a constitutively active *Card14* variant, orthologous to the human mutation, and develops spontaneous chronic psoriasiform disease. In the human component of this study (n=36 patients with psoriasis versus n=47 non-psoriatic controls), circulating CD4^+^ memory T cells producing IL-9 and carrying a Th9 transcriptomic signature were expanded in a subset of patients, and this expansion was significantly associated with high-risk atherosclerotic cardiovascular disease (ASCVD). In the murine models, these cells showed a tendency to migrate into coronary vessels, and T cells expressing the Th9 lineage-defining transcription factor PU.1 were identified within human atherosclerotic plaques from individuals with psoriasis (26). Consistent with a broader role of T cells in plaque immunity, single-cell profiling of human atherosclerotic plaques has documented distinct activated CD4^+^ T cell subsets (27). Germline deletion of *Il9R*, endothelial-specific deletion of *Il9R*, and treatment with neutralizing anti-IL-9 antibodies each reduced lipid-rich aortic plaque formation. Notably, the reduction in plaque occurred largely independently of changes in aortic immune cell infiltration. These findings highlight a proatherogenic immune mechanism that is not targeted by current biologic therapies highlighting the possibility that some psoriasis-associated cardiovascular risk may persist despite effective anti-IL-17 or anti-TNF-α treatment.

A genetic model supporting a contributory role of chronic skin inflammation in accelerated atherogenesis is the K14-Rac1V12 model, in which a constitutively active form of the small GTPase Rac1 is selectively expressed in basal keratinocytes under the transcriptional control of the keratin-14 promoter (28). This transgene reproduces the hyperactive Rac1 signaling documented in human psoriatic lesions and induces a chronic psoriasiform skin phenotype. Bone marrow-derived macrophages (BMDMs) from these mice exhibited a proatherogenic phenotype that was characterized by an increased lipid uptake, an enhanced foam cell formation with a ~6-fold increase in cholesterol crystal formation.

To study the cardiovascular consequences of this skin phenotype, K14-Rac1V12 mice were crossed into an atherosclerosis-prone background carrying deletion of scavenger receptor B 1 (Srb1−/−) combined with a hypomorphic ApoER61H/H allele. After two weeks of high-fat diet, psoriatic mice developed larger aortic plaques than littermate controls (6.8% versus 1.8% of aortic area), indicating that an epidermal-intrinsic defect is sufficient to accelerate atherogenesis. Since SOD2 had been implicated separately in keratinocyte hyperproliferation and in macrophage cholesterol homeostasis, the role of SOD2 in psoriatic macrophages was examined in the study. Both expression and enzymatic activity were found to be reduced, Sirt3 was downregulated and mitochondrial ROS accumulated. Restoring SOD2 activity with the mimetic MnTBAP normalized lipid uptake, foam cell formation and cholesterol crystal formation, identifying SOD2 as a modifiable regulator of the proatherogenic macrophage phenotype (28).

## Bidirectional amplification and multi-organ involvement

Emerging preclinical evidence suggests that the two pathologic conditions engage a reciprocal amplification loop in which pre-existing atherosclerosis can worsen psoriatic skin inflammation and *vice versa*. Single-cell RNA sequencing of skin biopsies from treatment-naïve patients with coexisting atherosclerosis, integrated with public atherosclerotic plaque transcriptomes, supported this view (29, 30). CD11b^+^ M1 macrophages were increased in psoriatic lesions from patients with atherosclerosis and shared transcriptomic signatures with macrophages in atherosclerotic plaque. The abundance of these macrophages correlated with the Psoriasis Area and Severity Index (PASI), and exposure of human monocyte-derived macrophages to recombinant GPIb *in vitro* induced the M1 activation marker CD86 in a dose-dependent manner. In the imiquimod model, pharmacological activation of CD11b with the allosteric agonist ADH-503 was sufficient to worsen dermatitis, supporting a bidirectional circuit in which atherosclerosis aggravates cutaneous inflammation through platelet-derived glycoprotein Ib (GPIb), the platelet adhesion receptor for von Willebrand factor. In the atherosclerotic context, GPIb engaged CD11b on monocyte-derived macrophages and drove their M1 polarization in a contact-dependent manner (29). The lipid-to-skin direction was confirmed in transgenic mice overexpressing human cholesteryl ester transfer protein (CETP) or phospholipid transfer protein (PLTP). These mice developed more severe psoriasiform lesions than wild-type controls upon imiquimod treatment (31). CETP-Tg and PLTP-Tg mice also showed increased epidermal proliferation (PCNA positivity) and elevated cutaneous mRNA levels of IL-17A, IL-17F, IL-22, IL-23p19, IFN-α, IL-1β and IL-6, together with raised plasma TNF-α. These results indicate that the proatherogenic dyslipidemia characteristic of psoriasis is not merely a downstream consequence of skin inflammation but can amplify the cutaneous inflammatory responses.

In a convergent line of evidence, PCSK9, a protein predominantly expressed in the liver that raises circulating LDL by promoting LDL-receptor degradation, was characterized in two separate human psoriasis cohorts (n = 88 and n = 20, each compared with age-, sex-, and cholesterol-matched healthy controls) and in the K14-Rac1V12-/+ murine model (32). Circulating PCSK9 levels were higher in patients with psoriasis than in matched controls and correlated with disease severity. The increase was associated with measures of subclinical atherosclerosis independently of conventional cardiovascular risk factors. Immunohistochemical staining showed that PCSK9 was overexpressed in psoriatic lesions, both in the K14-Rac1V12 mouse model and in human biopsies, without a parallel increase in hepatic expression. These findings identify PCSK9 as a skin-derived mediator associated with both early and advanced atherosclerosis. The systemic oxidative burden of psoriasiform inflammation extended beyond the vasculature to other organs. In a murine model, psoriasiform inflammation led to renal dysfunction through activation of NADPH oxidases and inducible nitric oxide synthase (33), while in a rat model it was associated with impaired myocardial function and increased cardiac oxidative stress (34), indicating that oxidative mechanisms, analogous to those operating in the vessel wall, may drive multiorgan pathology.

## Discrepancies among models and translational limitations

Despite the converging mechanistic evidence, a significant discrepancy exists regarding the potential of psoriasiform skin inflammation to accelerate atherosclerosis in atherosclerosis-prone mice. This discrepancy is primarily determined by the type of model employed.

In LDLR^−/−^ mice on a high-cholesterol diet, cyclic imiquimod application induced psoriasiform lesions and systemic inflammation, with splenomegaly and elevated plasma IL-17A. Despite this systemic activation, atherosclerosis was not accelerated: plaque formation was attenuated in the aortic arch and unchanged in the aortic root compared with non-IMQ controls (35). Furthermore, results obtained from the application of TPA on the ears of ApoE^−/−^ mice for 8 weeks showed that despite a measurable systemic inflammation no differences in the aortic plaque area or composition were observed (36). These negative results contrast with the ~7-fold acceleration of atherogenesis observed when the K14-Rac1V12 skin phenotype was combined with an atherosclerosis-prone background (28) and with the increased plaque formation in IMQ-ApoE^−/−^ mice on high-fat diet in the Th9 study (26).

This divergence reflects fundamental differences between the mouse models. The standard imiquimod protocol involves a short application period that activates Toll-like receptors 7/8 and triggers a predominantly innate, acute inflammatory response. This acute model does not reproduce the chronic immunopathology that characterizes human psoriasis and develops over months to years. By contrast, several genetic and spontaneous models (K14-Rac1V12, KC-Tie2, Kcasp1Tg, K14-IL-17Aind/+) are associated with accelerated atherogenesis. A shared feature of these models is a sustained psoriasiform inflammation, in some cases accompanied by a prothrombotic state, that more closely mirrors the prolonged disease course seen in humans (24, 28).

Although LL-37-reactive CD8^+^ effector memory T cells persist in the peripheral blood of patients with acute coronary syndrome, adoptive transfer of T cells from mCRAMP-immunised ApoE^−/−^ mice into ApoE^−/−^ recipients paradoxically reduced aortic plaque area by 28%. This protective effect was attributed to a skewing of the response towards an IL-10^+^/IFN-γ^+^ regulatory phenotype (37). Notably, a recent study raised a critical concern about the IMQ model claiming that results are context-dependent and vary with genetic background and application protocol (38). Despite the specific limitations of each model, a recurring observation across these studies is that models reproducing a sustained, chronic psoriasiform inflammation tend to display a vascular phenotype, whereas models limited to acute, innate-driven inflammation generally do not. This pattern is consistent with a contributory role of inflammatory chronicity, although the available models differ in genetic background and inflammatory drivers as well as in duration, and these variables cannot be fully distinguished.

## Perivascular and epicardial adipose tissue as a converging compartment

A further intersection between psoriasis and the cardiovascular compartment involves the inflammatory expansion of perivascular and epicardial adipose tissues (EAT) and their potential role as an anatomical site where the above mechanisms converge. In male patients with severe psoriasis, epicardial adipose tissue volume was greater than in risk factor-matched controls, in the absence of overt coronary disease or diabetes and with no difference in body mass index between groups (39). A recent imaging review has similarly summarized evidence that epicardial and pericoronary adipose tissue (PCAT) is expanded in psoriasis and may be modulated by systemic therapy (40), although a direct demonstration that these compartments integrate the proposed mechanisms in psoriasis is still lacking. Inflammation within this depot has been hypothesized to contribute to the distinctive pattern of cardiovascular involvement seen in psoriasis (41).

In patients with atrial fibrillation, EAT was enriched in transcriptionally distinct CD8^+^ tissue-resident memory T cells (T_RM_) that infiltrated the EAT-myocardial interface and altered cardiomyocyte calcium handling in coculture (42). Although not specifically demonstrated in psoriasis, T_RM_ persistence in peripheral tissues depended on exogenous lipid uptake (43), suggesting that the inflammation-driven expansion of perivascular and epicardial fat may provide a permissive niche for skin-primed T cell effectors. Cutaneous adipose tissue itself carried a strong IL-17-dependent inflammatory signature in patients with psoriasis, partially reversed by IL-17A blockade (44). Dermal adipose tissue macrophages were identified as a source of pro-platelet basic protein (PPBP), which exacerbated atherosclerosis in ApoE^−/−^ mice by inducing mitochondrial dysfunction in human coronary artery endothelial cells (45). In obese mice, systemic inflammation drove pathological remodeling of visceral adipose tissue, which in turn exacerbated psoriasiform inflammation, pointing to a reciprocal amplification loop between the cutaneous lesions and the systemic adipose compartment in the context of metabolic dysfunction (46).

## From mouse to human: the translational concept

**Figure 2:**
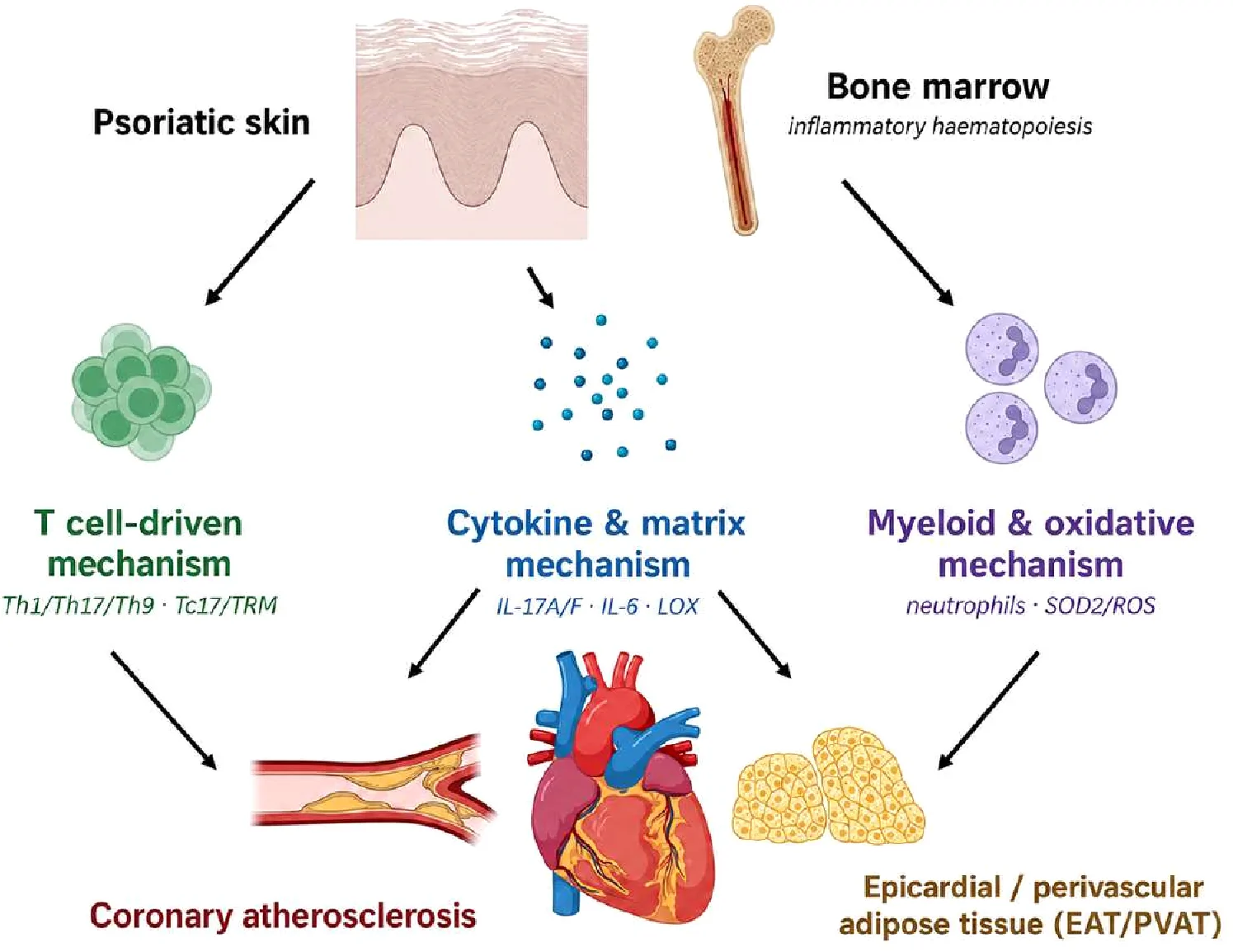
Graphical representation of the three converging mechanisms that carry psoriatic skin inflammation to the cardiovascular compartment. Original figure created by Biorender.com.

The mouse studies identified three possible psoriasis-driven mechanistic links with cardiovascular disease: (i) a cytokine/matrix-remodeling axis (16, 19), (ii) a T-cell-driven proatherogenic mechanism (21, 26) and (iii) a myeloid and oxidative mechanism (28). Rather than treating these pathways individually, the available human data are interpreted as constituent parts of a single, converging architecture (**Figure 2**). Within this framework, the perivascular and epicardial adipose compartments serve as the proposed anatomical sites where these mechanisms integrate.

## Subclinical vascular disease and cardiac involvement: imaging evidence

In patients with psoriasis, the qualitative features of coronary plaque display an imaging signature which is consistent with the murine-defined IL-17/LOX/lipoprotein entrapment axis. Direct CCTA studies confirmed enrichment in lipid-rich necrotic core (LRNC) and non-calcified components of coronary plaque in patients with psoriasis (9). Direct CCTA confirmed that patients with psoriasis carry a greater non-calcified and high-risk plaque burden than their traditional risk profile would predict (47). Plaque prevalence is nearly doubled in psoriatic arthritis compared with risk factor-matched controls (48, 49). Whether this excess reflects a causal effect of skin inflammation or a shared susceptibility between psoriasis and atherosclerosis remains debated (8, 50). Disease duration was associated with both vascular inflammation and MACE, with the risk of cardiovascular events rising by approximately 1% for each additional year of disease (12).

This evidence is consistent with the murine observation that sustained inflammation is associated with the cardiovascular phenotype. Indeed, in patients, both the severity and the systemic burden of psoriatic inflammation have been linked to aortic vascular inflammation detected by ^18^FDG-PET/CT (51, 52). The skin-vessel link is pharmacologically modifiable: in a cohort of 115 patients with psoriasis followed for one year, a median 33% improvement in PASI was paralleled by a 6% reduction in aortic target-to-background ratio on ^18^F-fluorodeoxyglucose positron emission tomography/computed tomography (^18^F-FDG PET/CT) (53). Across observational cohorts of biologic-naïve patients, one year of biologic therapy reduced non-calcified coronary burden (54), lipid-rich necrotic core (55, 56), and the perivascular fat attenuation index, a computed tomography (CT)-derived marker of coronary inflammation (57). In the murine models, the same IL-17-driven vascular changes regressed with anti-IL-17 treatment and with inhibition of lysyl oxidase by β-aminopropionitrile (BAPN) (15, 16, 19).

Beyond the coronary tree, an imaging-defined subclinical vascular signature was already detectable in patients with recent-onset psoriatic arthritis without traditional risk factors, combining increased carotid intima-media thickness with elevated serum levels of ICAM-1, IL-6, MCP-1 and CRP (58).

At the microvascular level, a reduced coronary flow reserve on doppler echocardiography was reported in approximately one-third of asymptomatic patients with severe psoriasis, with disease severity and duration as independent predictors (59). Another study showed that circulating endothelial progenitor cells were reduced in inverse proportion to arterial stiffness (60).

Furthermore, in a study with 39 patients with psoriatic arthritis, ^18^F-FDG uptake was elevated in bone marrow, spleen, liver and subcutaneous adipose tissue.

In addition, visceral adipose tissue volume correlated with total coronary plaque burden, accompanied by elevated circulating levels of high-sensitivity C-reactive protein (hs-CRP), IL-6 and IFN-γ (61).

## Inflammatory hematopoiesis, lipoprotein dysfunction, and the circulating biomarker landscape

Vascular inflammation in psoriasis is not confined to the vessel wall but embedded in a multi-systemic inflammatory state involving the spleen, the bone marrow and the adipose tissue, providing the human counterpart of the inflammatory hematopoiesis identified in K14-IL-17A photoconversion experiments (20).

In a study with 240 participants (210 with psoriasis and 30 healthy controls), splenic and bone marrow ^18^F-FDG uptake independently predicted non-calcified coronary burden and lipid-rich necrotic core (62), while visceral adiposity correlated with aortic vascular inflammation independently of cardiometabolic factors (63).

Chronic systemic inflammation expands visceral adipose tissue and accelerates non-calcified coronary burden over time, with bone marrow ^18^F-FDG uptake linking visceral fat to lipid-rich necrotic core. In male patients however subcutaneous adipose tissue was negatively associated with noncalcified and lipid-rich necrotic core burden (64–66).

At the lipoprotein level, reduced ApoA-I and a shift towards small dense and oxidized lipoproteins track plaque burden (67) (68), with LL-37 bridging between skin and vessel (69).

In this environment, circulating monocytes display an altered, proinflammatory phenotype, with upregulation of CXCL16 in patients with concomitant metabolic syndrome (70). Metabolic syndrome and its individual components are in turn associated with a higher non-calcified coronary burden (71, 72).

A panel of circulating mediators mirrors the three axes previously described. In this context GlycA (a nuclear magnetic resonance-derived composite glycoprotein inflammatory marker) and CCL20 represent the cytokine and chemokine compartment, soluble LOX-1 the oxidative endothelial branch, and the alarmin S100A8/A9 the myeloid axis. S100A8/A9 is reduced by biologic therapy in proportion to plaque regression, while peripheral myeloid indices recapitulate the inflammatory hematopoiesis observed in the mouse (73–79). The cytokine and myeloid axes are the best translated, extending to peripheral blood transcriptomic signatures proposed as predictors of treatment response (80).

## Antipsoriatic treatment and cardiovascular outcomes: consistent signals and unresolved endpoints

Observational evidence has consistently suggested that an effective antipsoriatic treatment translates into a measurable cardiovascular benefit. In psoriatic disease, TNF inhibitors slowed the progression of carotid plaques (81). In a large registry analysis, treatment with biologics was associated with lower all-cause mortality and a reduced risk of major adverse cardiovascular events compared with conventional antipsoriatic drugs (82).

Moreover, TNF blockade restored coronary microvascular function in patients with severe psoriasis treated with TNF-α inhibitors (83).

Similarly, in a three-arm study of 150 patients (secukinumab versus ciclosporin versus methotrexate), anti-IL-17A treatment produced a greater 12-month improvement in left ventricular global longitudinal strain (a 14% increase, versus 2% with ciclosporin and 4% with methotrexate), coronary flow reserve and pulse wave velocity, whereas ciclosporin worsened arterial stiffness (84).

In murine models, the vascular consequences of IL-17 signaling proved to be reversible. Anti-IL-17 treatment restored vascular function in CD11c-IL-17Aind/ind model, and the inhibition of lysyl oxidase with β-aminopropionitrile (BAPN) reduced the entrapment of lipoproteins in the arterial wall (15, 16). Moreover, in patients with psoriatic arthritis, the achievement of minimal disease activity had a protective effect on the progression of subclinical atherosclerosis and arterial stiffness (85).

The observation that effective antipsoriatic treatment translates into a measurable cardiovascular benefit was also tested in the Vascular Inflammation in Psoriasis (VIP) program. The trials measured aortic vascular inflammation on ^18^F-FDG PET/CT. None of the agents met that endpoint: neither adalimumab nor phototherapy (86), secukinumab (87) nor apremilast (88) significantly reduced aortic vascular inflammation between 12 and 52 weeks. This apparent discrepancy may reflect, the relatively short duration of these trials, which could be insufficient to reverse a vascular phenotype that develops over years. A study drawing on two independent resources associated longer psoriasis duration with increased cardiovascular risk. In an imaging cohort (n = 190), longer disease duration correlated with greater aortic vascular inflammation on ^18^F-FDG PET/CT. Separately, in a nationwide registry (n = 87,161), each additional year of disease was associated with an approximate 1% increase in major adverse cardiovascular events (12). The observation would also be in line with the preclinical observation that murine models sustaining chronic inflammation reproduce a vascular phenotype that acute models do not (26).

Standard risk calculators misclassify as low risk many psoriasis patients who already carry high-risk coronary plaque (11). Prevention must therefore integrate the temporal dimension of the disease with residual risk determinants that are not adequately addressed by cytokine-targeted therapy, including chronic psychological stress, lipoprotein(a) and circulating signatures associated with cardiovascular events (89–92).

## Conclusion

The cardiovascular phenotype of psoriasis is not the product of a single mechanism but of three converging mechanisms that carry skin-derived inflammation to the vessel wall: a cytokine/matrix-remodeling axis driven by IL-17A and IL-17F, a T-cell-driven mechanism in which skin-primed effector cells act on the vascular endothelium, and a myeloid-oxidative mechanism fueled by systemic, inflammatory hematopoiesis. Inflammatory chronicity of inflammation, rather than the activity of any single mediator, represents the key determinant of this cardiovascular phenotype. These mechanisms in humans still need to be confirmed, even if the myeloid and cytokine pathway are broadly supported by imaging, biomarker and treatment-response data. The perivascular and epicardial adipose tissue, expanded in association with chronic inflammation, represents a candidate anatomical site where these mechanisms may converge. Traditional risk factors usually underestimate cardiovascular risk in psoriatic disease and should evolve towards a chronicity-aware, mechanism-based cardiovascular risk algorithm. This prediction algorithm should incorporate subclinical vascular imaging, metabolic and oxidative drivers to optimize early intervention and prevent adverse cardiovascular outcomes.
