# Supplementary material for: From skin to heart: a state-of-the-art perspective on psoriasis-driven cardiovascular comorbidities
Source: Front Immunol. 2026 Sep 9;17:1923150. doi: 10.3389/fimmu.2026.1923150 (PMC13598789; doi:10.3389/fimmu.2026.1923150)
Supplement: Supplementary file 1 [file Table1.docx]

**Supplementary Table S1. Original studies discussed in this review, grouped by thematic category.**

| **Ref.** | **First author, Year** | **Study type** | **Model / Design** | **Key findings** |
| --- | --- | --- | --- | --- |
| **Preclinical mechanistic models (n = 18)** | | | | |
| **15** | Huang LH, 2019 | Preclinical | IMQ C57BL/6 mouse | IL-17 induces LOX-mediated interstitial entrapment of HDL and LDL in skin and aorta; reversed by anti-IL-17 or by BAPN. |
| **16** | Schüler R, 2019 | Preclinical | Models of graded severity: two IL-17A-overexpressing lines (K14-IL-17A; CD11c-IL-17A) and the acute IMQ model | Serum IL-17A concentration tracks the severity of vascular dysfunction across the three models; anti-IL-17A improves endothelial function. |
| **19** | Nakanishi T, 2024 | Preclinical | Kcasp1Tg crossed with IL-17A/F KO | Aortic arteriosclerotic remodelling requires deletion of both IL-17A and IL-17F, indicating a non-redundant contribution of IL-17F beyond IL-17A; the cellular source of IL-17F was not directly determined. |
| **20** | Schaller T, 2023 | Preclinical | K14-IL-17Aind/+ × PhAM mouse (photoconversion cell tracking) | Pro-inflammatory neutrophils reaching the aortic wall originate from the bone marrow, not from the psoriatic skin; ROS-mediated vascular inflammation. |
| **21** | Casciano F, 2025 | Preclinical + human PB phenotyping | Recurrent IMQ C57BL/6 mouse + 36 psoriasis patients | Repeated IMQ cycles expand CXCR3+ highly differentiated memory T cells systemically; aortic Icam1, Vcam1, Ccl2 and Olr1 upregulated. |
| **24** | Wang Y, 2016 | Preclinical | KC-Tie2 mouse with or without IL-6 KO | Genetic deletion of IL-6 restores thrombotic occlusion time towards control levels without altering skin score, identifying skin-derived IL-6 as a critical link to the pro-thrombotic state. |
| **26** | Baral I, 2025 | Preclinical + human cohort | IMQ-ApoE−/−, IL-23-ApoE−/− and Card14ΔE138-ApoE−/− mouse on HFD; human ASCVD cohort | Th9/IL-9R/STAT3 axis acts directly on arterial endothelium; chronic Card14 model reproduces atherosclerosis whereas acute IMQ does not; anti-IL-9 reduces aortic plaque area; expanded Th9 cells associate with ASCVD in patients. |
| **28** | Baumer Y, 2018 | Preclinical | K14-Rac1V12 × ApoE−/− × HFD mouse | SOD2 expression reduced by approximately 60% in psoriasis macrophages; mitochondrial ROS accumulate and drive foam cell formation; atherogenesis accelerated approximately 7-fold; restoration of SOD2 activity reverses the pro-atherogenic phenotype. |
| **29** | Dong C, 2025 | Preclinical (scRNA-seq + mouse) | Patient skin scRNA-seq integrated with IMQ mouse | Platelet GPIb upregulated in psoriatic skin in a perivascular distribution, correlated with PASI; engages monocyte CD11b to drive M1 polarisation; bidirectional skin-atherosclerosis circuit. |
| **31** | Chen J, 2022 | Preclinical | CETP-Tg or PLTP-Tg IMQ mouse | Pro-atherogenic dyslipidaemia worsens psoriasiform skin inflammation; supports a lipid-to-skin direction of the bidirectional axis. |
| **32** | Garshick MS, 2021 | Preclinical + clinical | Psoriasis cohort with K14-Rac1V12 mouse confirmation | PCSK9 overexpressed in psoriatic skin but not in liver; circulating PCSK9 correlates with disease severity and with subclinical atherosclerosis. |
| **33** | Al-Harbi NO, 2017 | Preclinical | IMQ mouse, renal endpoints | Psoriasis-like inflammation induces NOX2, NOX4 and iNOS upregulation with renal dysfunction; reversed by N-acetylcysteine or by NOX/iNOS inhibition. |
| **34** | Corbic M, 2025 | Preclinical | IMQ rat with isolated heart preparation | Cardiac contractility and relaxation impaired; myocardial oxidative stress increased; cardiac collagen deposition increased several-fold. |
| **35** | Madsen M, 2018 | Preclinical | Cyclic IMQ in LDLR−/− mouse on high-cholesterol diet | Psoriasiform lesions and systemic inflammation induced, yet atherosclerosis not accelerated; aortic arch plaque formation attenuated. |
| **36** | Madsen M, 2016 | Preclinical | TPA in hypercholesterolaemic ApoE−/− mouse | Cutaneous TPA application produces systemic inflammation but no acceleration of atherosclerotic plaque. |
| **37** | Chernomordik F, 2020 | Preclinical (+ human ACS PB) | ApoE−/− adoptive transfer + acute coronary syndrome patients | LL-37-reactive CD8+ effector memory T cells persist in ACS patients; transfer of T cells from mCRAMP-immunised mice paradoxically reduces aortic plaque area and abolishes calcification. |
| **38** | Smith CC, 2026 | Preclinical critical re-analysis | Systematic re-evaluation of the IMQ model | Acute IMQ produces an inconsistent and context-dependent cardiovascular phenotype across studies; chronicity identified as the missing variable for translational validity. |
| **69** | Nakamura Y, 2024 | Translational (human + mouse) | Human monocyte-derived macrophages in vitro + mouse atherosclerosis | LL-37 enhances LDL uptake by human macrophages via LDLR, SR-B1 and CD36; recombinant LL-37 accelerates plaque formation in murine atherosclerosis. |
| **Coronary plaque imaging (CCTA and calcium scoring) (n = 7)** | | | | |
| **11** | Gonzalez-Cantero A, 2022 | Clinical observational, two cohorts | Two independent psoriasis cohorts with CCTA and risk-score comparison | Standard cardiovascular risk algorithms underperform: a substantial proportion of patients classified as low-risk carry high-risk imaging-defined coronary plaque. |
| **47** | Lerman JB, 2017 | Clinical observational | Prospective CCTA cohort with biologic intervention | Coronary plaque enriched in lipid-rich necrotic core and non-calcified components; high-risk features improve after treatment. |
| **48** | Shen J, 2017 | Clinical observational | CCTA in psoriatic arthritis vs risk factor-matched controls | Coronary plaque prevalence nearly doubled in psoriatic arthritis compared with controls, without prior diagnosis of coronary disease. |
| **65** | Manyak GA, 2022 | Clinical observational | Cross-sectional psoriasis cohort + mediation analysis | Abdominal visceral adipose tissue associates with lipid-rich necrotic core; mediation model identifies bone marrow 18F-FDG uptake as a partial statistical intermediate. |
| **71** | Teklu M, 2021 (JAAD) | Clinical observational | Psoriasis cohort with CCTA | Metabolic syndrome and its individual components associate with non-calcified coronary burden in psoriasis. |
| **74** | Florida EM, 2023 | Clinical longitudinal | Psoriasis cohort with serial CCTA | Soluble LOX-1 tracks coronary plaque progression over time. |
| **77** | Dey AK, 2021 | Clinical observational | Psoriasis cohort with CCTA | Neutrophil-to-lymphocyte ratio associates with non-calcified coronary burden. |
| **Vascular inflammation imaging (FDG-PET) (n = 4)** | | | | |
| **49** | Joshi AA, 2018 | Clinical observational | FDG-PET/CT and CCTA in psoriasis cohort | Aortic vascular inflammation associates with coronary plaque characteristics. |
| **61** | Schwartz DM, 2022 | Clinical observational | Psoriatic arthritis cross-sectional, FDG-PET/CT + CCTA | 18F-FDG activity elevated in bone marrow, spleen, liver and subcutaneous adipose tissue, each correlating independently with non-calcified coronary burden; visceral adipose tissue an additional independent correlate. |
| **62** | Patel NH, 2021 | Clinical observational | Psoriasis cohort, FDG-PET/CT + CCTA | Splenic and bone marrow 18F-FDG uptake independently predict non-calcified coronary burden and lipid-rich necrotic core after adjustment for cardiovascular risk factors. |
| **73** | Joshi AA, 2016 | Clinical observational | Psoriasis cohort with NMR spectroscopy and PET/CT | GlycA, a composite NMR signal of acute-phase glycoproteins, associates with aortic vascular inflammation and with non-calcified coronary burden. |
| **Microvascular and myocardial function; carotid imaging (n = 3)** | | | | |
| **58** | Kolliker Frers RA, 2018 | Clinical observational | Recent-onset psoriatic arthritis without conventional CV risk factors | Subclinical vascular signature including increased cIMT and elevated sICAM-1, IL-6, MCP-1 and CRP detectable before traditional risk algorithms identify patients as high-risk. |
| **59** | Piaserico S, 2023 | Clinical observational | Doppler echocardiography in severe psoriasis | Coronary flow reserve reduced in approximately one-third of asymptomatic patients; severity and duration emerge as independent predictors. |
| **60** | Liu JH, 2016 | Clinical observational | Psoriasis cohort | Circulating endothelial progenitor cells reduced and inversely correlated with arterial stiffness. |
| **Adipose tissue imaging (n = 3)** | | | | |
| **63** | Rivers JP, 2018 | Clinical observational | Psoriasis cohort, FDG-PET/CT | Visceral adiposity associates with aortic vascular inflammation independently of cardiometabolic risk factors. |
| **64** | Sajja A, 2020 | Clinical longitudinal | Psoriasis cohort, 4-year follow-up | Persistently elevated hs-CRP is accompanied by greater visceral adipose volumes and steeper progression of non-calcified coronary burden than persistently low hs-CRP; chronic systemic inflammation acts as a temporal driver of the adipose-coronary axis. |
| **66** | Teklu M, 2021 (Am J Prev Cardiol) | Clinical observational | Psoriasis cohort, sex-stratified | Abdominal subcutaneous adipose tissue negatively associates with non-calcified and lipid-rich necrotic core burden in men; depot-specific contribution to cardiovascular phenotype. |
| **Treatment effects on vascular endpoints (n = 13)** | | | | |
| **53** | Dey AK, 2017 | Clinical interventional, observational design | PET/CT case-cohort, 1-year follow-up | Median 33% PASI improvement paralleled by median 6% aortic target-to-background ratio reduction over one year of treatment. |
| **54** | Elnabawi YA, 2019 (Cardiovasc Res) | Clinical observational | Biologic-naïve psoriasis cohort, 1-year follow-up | Non-calcified coronary burden decreases after one year of biologic therapy in biologic-naïve psoriasis. |
| **55** | Choi H, 2020 | Clinical observational | Biologic-naïve psoriasis cohort, 1-year follow-up | Lipid-rich necrotic core regresses after one year of biologic therapy, independently of the class of agent used. |
| **57** | Elnabawi YA, 2019 (JAMA Cardiol) | Clinical observational | Propensity-matched psoriasis cohort, 1-year follow-up | Perivascular fat attenuation index declines significantly in biologic-treated patients (P<0.001); unchanged in untreated controls (P=0.95). |
| **75** | Berg AR, 2022 | Clinical observational | Psoriasis cohort with biologic therapy | S100A8/A9 associates with lipid-rich necrotic core and declines under biologic therapy in proportion to plaque regression. |
| **81** | Eder L, 2018 | Clinical observational | Psoriatic disease cohort | TNF inhibitor treatment slows the progression of carotid plaque. |
| **82** | Kridin K, 2025 | Clinical, large registry | International registry analysis | Biologic therapy mitigates the risk of death and major adverse cardiovascular events compared with classic antipsoriatic drugs or apremilast. |
| **83** | Piaserico S, 2016 | Clinical observational | Young severe-psoriasis cohort | TNF inhibitor restores coronary microvascular function in patients with severe psoriasis. |
| **84** | Makavos G, 2020 | Clinical, three-arm comparative | 150 psoriasis, secukinumab vs ciclosporin vs methotrexate, 12 months | Anti-IL-17A improves 12-month left ventricular global longitudinal strain (14% versus 2-4%), coronary flow reserve and pulse wave velocity more than ciclosporin or methotrexate; ciclosporin worsens arterial stiffness. |
| **85** | Cheng IT, 2019 | Clinical prospective | Psoriatic arthritis cohort | Achievement of sustained minimal disease activity has a protective effect on the progression of subclinical atherosclerosis and arterial stiffness. |
| **86** | Mehta NN, 2018 (VIP) | RCT, double-blind, placebo-controlled | Adalimumab vs narrow-band UVB vs placebo, 12 weeks | Neither adalimumab nor phototherapy significantly reduces aortic vascular inflammation compared with placebo at the 12-week primary endpoint. |
| **87** | Gelfand JM, 2020 (VIP-S) | RCT, double-blind, placebo-controlled | Secukinumab vs placebo, 12-week endpoint + 52-week extension | Aortic TBR difference −0.053 (95% CI −0.169 to 0.064; P=0.37) at 12 weeks; no improvement through 52 weeks. |
| **88** | Gelfand JM, 2022 (VIP-A) | Open-label, single-arm | Apremilast 30 mg twice daily, 16-week endpoint + 52 weeks | Aortic vascular inflammation neutral (TBR −0.02; 95% CI −0.08 to 0.05; P=0.61); visceral and subcutaneous adipose tissue reduced approximately 5-6%; ApoA-I increased. |
| **Circulating biomarkers, cardiometabolic risk and comorbidities (n = 14)** | | | | |
| **10** | Compagnucci P, 2025 | Clinical observational | Multicentre psoriasis-AF cohort | Catheter ablation of atrial fibrillation in patients with psoriasis: procedural outcomes and clinical characteristics described in a multicentre setting. |
| **12** | Egeberg A, 2017 | Clinical observational, two cohorts | NIH FDG-PET/CT cohort (vascular inflammation, n=190) and Danish nationwide registry (MACE, n=87,161) | Psoriasis duration associates with vascular inflammation in the imaging cohort and, independently, with major adverse cardiovascular events in the registry (approximately 1% increase in event risk per additional year of disease); the two outcomes were assessed in separate cohorts and were not directly linked. |
| **67** | Teague HL, 2023 | Clinical observational | Psoriasis cohort | Circulating ApoA-I reduced and inversely correlated with initiation and progression of atherosclerosis. |
| **68** | Sorokin AV, 2018 | Clinical observational | Psoriasis cohort with CCTA | Oxidation-modified lipoproteins and small dense LDL enriched in serum; small dense LDL correlates with coronary plaque burden. |
| **70** | Schielke L, 2022 | Clinical observational | Psoriasis with or without metabolic syndrome | Monocyte CXCL16 upregulation and innate lymphoid cell imbalance amplified by concomitant metabolic syndrome. |
| **72** | O'Hagan R, 2023 | Clinical observational | Psoriasis cohort | Triglyceride-glucose index correlates with insulin resistance and with subclinical atherosclerosis. |
| **76** | Elnabawi YA, 2021 | Clinical observational | Psoriasis cohort | CCL20 correlates with disease severity and with impaired vascular health. |
| **78** | Berna-Rico E, 2024 | Clinical observational, two cohorts | Two independent psoriasis cohorts | Monocyte-to-HDL ratio associates with systemic inflammation, insulin resistance and subclinical coronary atherosclerosis. |
| **79** | Teague HL, 2019 | Clinical observational | Psoriasis cohort | Distinct neutrophil subsets and platelet activation associate with vascular disease. |
| **80** | Cantó E, 2025 | Clinical translational | Psoriasis transcriptomic study | Peripheral blood transcriptomic immune hubs proposed as candidate predictors of therapeutic response to biologic agents. |
| **89** | Aberra TM, 2016 | Clinical observational | Psoriasis cohort | Self-reported depression in psoriasis associates with subclinical vascular disease. |
| **90** | Lateef SS, 2020 | Clinical longitudinal | Psoriasis cohort with neuroimaging | Chronic stress-related neural activity associates with physiological dysregulation and with coronary artery disease. |
| **91** | Alebna PL, 2025 | Clinical observational | Autoimmune conditions cohort | Lipoprotein(a) associates with cardiovascular events in individuals with autoimmune conditions, including psoriasis. |
| **92** | Colaco K, 2021 | Clinical prospective | Psoriatic arthritis and psoriasis cohort with metabolomics | Targeted metabolomic profiling predicts cardiovascular events in psoriatic arthritis and psoriasis. |

*Reference numbers follow the manuscript bibliography (version 29-07). Studies are grouped by their primary focus; studies whose primary aim was to assess the effect of a treatment on vascular endpoints are listed under “Treatment effects on vascular endpoints” irrespective of the imaging modality used. Original studies only; reviews, editorials and hypothesis papers cited for context are not included.*
